# End-tidal carbon dioxide changes induced by passive leg raising can predict fluid responsiveness in patients on veno-arterial extracorporeal membrane oxygenation: a prospective, interventional study

**DOI:** 10.1186/s13613-025-01604-2

**Published:** 2025-11-20

**Authors:** Timothée Hannequin, Aurore Ughetto, Jacob Eliet, Cinderella Blin, Aurélien Canon, Marine Saour, Philippe Gaudard, Celia Vidal, Nicolas Molinari, Pascal Colson, Marc Mourad

**Affiliations:** 1 Department of Anesthesiology and Critical Care Medicine, Arnaud de Villeneuve Hospital, CHRU Montpellier, 371 Avenue du Doyen Gaston Giraud, Montpellier, 34295 France; 2 CNRS, Inserm, PhyMedExp, Univ Montpellier, Montpellier, France; 3 IDESP, Inserm, PreMEdical INRIA, CHU Montpellier, Univ Montpellier, Montpellier, France; 4 Groupe Adène, Montpellier, France; 5 Institut de Génomique Fonctionnelle, CNRS, Inserm, Univ Montpellier, Montpellier, France

**Keywords:** End-tidal carbon dioxide, Volume expansion, Fluid responsiveness, Passive leg raising, Veno-arterial extracorporeal membrane oxygenation

## Abstract

**Background:**

Veno-arterial extracorporeal membrane oxygenation (VA-ECMO) is increasingly used in patients with cardiogenic shock. It results in cardiopulmonary shunting with reduced native cardiac output. Volume expansion is usually administered to increase native cardiac output, in order to improve peripheric perfusion or to avoid thromboembolic complications in cardiac cavities. End-tidal carbon dioxide (EtCO_2_) is known to be related to native cardiac output. Our hypothesis was that EtCO_2_ changes induced by passive leg raising predict fluid responsiveness in patients under VA-ECMO.

**Methods:**

In this prospective, interventional study, patients under VA-ECMO support were included, provided they required volume expansion. The protocol included three sequential steps: (1) Baseline in supine position (2) Passive leg raising (3) Volume expansion in basal position. Hemodynamic parameters were recorded at each step. Fluid responsiveness was defined as a velocity time integral at the left ventricle outflow tract increase of 15% or more after volume expansion. The ability of passive leg raising induced changes in EtCO_2_ to predict fluid responsiveness was evaluated with area under the receiver operating characteristics curve (AUC).

**Results:**

41 patients were studied; 58 passive leg raising - volume expansion tests were recorded. Fluid responsiveness was observed in 38 of the 58 measurements (65%). Passive leg raising test has correctly mimicked volume expansion (intraclass correlation coefficient 0.83 [0.73–0.9]). Considering all measurements, AUC of passive leg raising-changes in velocity time integral to predict fluid responsiveness was 0.89 [0.79–0.99] and remained good whatever the basal native cardiac output. Sensitivity and specificity were 92% [85–100] and 80% [69–90] respectively for a threshold of 15%. AUC of passive leg raising-induced changes in EtCO_2_ for predicting fluid responsiveness was good (0.98 [0.95-1]) only when basal native cardiac output was ≤ 1 L/min.

**Conclusion:**

Passive leg raising test can predict fluid responsiveness under VA-ECMO. EtCO_2_ changes induced by passive leg raising test can be used to detect fluid responsiveness in patients with native cardiac output ≤ 1 L/min under VA-ECMO. This test is easy to perform, reliable and may help to avoid unnecessary and potentially harmful fluid loading.

**Supplementary Information:**

The online version contains supplementary material available at 10.1186/s13613-025-01604-2.

## Introduction

Although recent randomized clinical trials did not shown a significant clinical benefit, peripheral veno-arterial extracorporeal membrane oxygenation (VA-ECMO) is increasingly used in patients with cardiogenic shock [1–3]. VA-ECMO operates by diverting blood from the right atrium towards the aorta, generating a pump outflow (PO), leaving a residual venous return to the right ventricle and the pulmonary circulation, or so-called native cardiac output (NCO). The total systemic output (TSO) results then from adding up NCO and PO, and should be adjusted to ensure adequate perfusion of organs while preserving NCO to avoid blood stasis in the cardiac cavities and aortic root [4, 5]. Volume expansion (VE) is one among various therapeutic to increase NCO, either to adjust TSO or to prevent thromboembolic complications before considering left ventricle venting techniques [6–8]. However, inappropriate VE can lead to fluid overload, which carries an increased risk of mortality [9, 10]. Excessive VE may also induce left ventricle distension and pulmonary oedema, a typical complication of VA-ECMO.

Prediction of fluid responsiveness (FR) appears then crucial under VA-ECMO. Validated tests to predict VE are based on dynamic indices but are dependent on many restrictive conditions. Many factors (low arterial pulsatility, low volume ventilation, cardiac arrhythmia) make these tests unsuitable during VA-ECMO support. Alternative tests have been proposed with postural manoeuvres that mimic a VE, such as the Trendelenburg or the passive leg raising (PLR) tests [11, 12]. Recently, Luo et al. demonstrated that a Trendelenburg manoeuvre was predictive of FR during VA-ECMO support [13]. However, that study excluded patients presenting a pulse pressure (PP) inferior to 15mmHg, so their results cannot be extrapolated to low pulsatility situations, often seen at the initial phase of VA-ECMO support and/or in case of the most severe cardiac dysfunctions. The PLR test has been studied under veno-venous ECMO [14] but, to our knowledge, not yet under VA-ECMO. To assess postural-induced changes in left ventricular stroke volume, echocardiography Doppler measurement of the systolic flow through the aortic valve with velocity-time integral (VTI) is a key issue [15]. However, VTI catching can be uneasy and time consuming in the context of very low cardiac output. In a previous study, we had observed that end-tidal carbon dioxide (EtCO_2_) was strongly correlated with NCO when it was lower than 2 L/min during VA-ECMO [16].

Therefore, we designed a prospective interventional study to assess the performance of PLR induced changes in EtCO_2_ to predict FR in VA-ECMO assisted patients.

## Methods

### Study design and setting

This is a prospective, single-centre, interventional study, approved by our institutional review board (Montpellier University Hospital; IRB: MTP-2020-01-201900314 University). Informed consent was obtained from all patients or their surrogates. The study was registered on Clinicaltrials.gov on July 21st, 2021 (NCT04984603). The study was conducted in an intensive care unit (ICU) of a tertiary University hospital, from February 2020 to April 2022.

### Characteristics of participants

All adults’ patients under VA-ECMO and invasive mechanical ventilation requiring VE were eligible. VE could be studied at any time of VA-ECMO support and several VEs for the same patient could be included only if separated by a 12-hours interval. VE indication followed our local institution’s protocol for managing low TSO and low NCO during VA-ECMO (Additional files, Figure S1). Exclusion criteria included age less than 18 years, pulmonary disorders (obstructive pulmonary disease, acute respiratory distress syndrome, cardiogenic shock due to massive pulmonary embolism), aortic insufficiency (≥ grade II), intra-cardiac shunt (atrial or ventricular communication), transient or durable left ventricle assist device, intra-aortic balloon pump support, evidence of hypovolemia with cannula suction and/or active bleeding, inability to generate reliable transthoracic cardiac echography, and unavailability of the research team for inclusion.

### Patient management

All patients were sedated with propofol, sufentanyl and/or dexmedetomidine. Continuous blood pressure was monitored via a radial artery catheter. Lung ventilation was managed with low respiratory rate (10–14 breaths/min) and tidal volume (4–6 mL/kg), and with positive end-expiratory pressure (8–10 cmH2O) to ensure protective ventilation. VA-ECMO consisted of polyvinyl chloride tubing with a membrane oxygenator (PH.I.S.I.O; Sorin Group, Italy), a centrifugal pump (Stockert scpc; Sorin Group, Italy), and percutaneous or surgically-inserted arterial and venous femoral cannulas (Optisite, Edwards Life-sciences, Germany and Venous HLS cannula, Getinge group, Germany) with an additional 7 F cannula (Super Arrow-Flew; Teleflex, Ireland) inserted distally into the femoral artery. An oxygen-air blender (Sechrist Industries, USA) ventilated the membrane oxygenator with a prespecified ventilation-to-perfusion ratio (V/Q) of 1 at implantation. Respiratory minute ventilation and ECMO sweep gas flow were then adjusted to maintain baseline PaCO_2_ in a normal range, around 40 mmHg. EtCO_2_ was measured from exhaled breath on a ventilator circuit and monitored using a ventilator CO_2_ analyser. Unfractionated heparin was administrated to maintain an anti-Xa activity between 0.2 and 0.3 IU/mL.

VA-ECMO flow was adjusted to the lowest rate necessary to ensure adequate tissue perfusion while maximizing NCO. The decision for VE followed our institution’s protocol (Additional files, Figure S1). In brief, VE could be proposed if TSO was deemed too low (hypotension defined as MAP < 65mmHg and signs of hypoperfusion (skin mottling, oligoanuria defined as diuresis < 0.5 mL/kg/h, lactate >2.2mmol/L)) or if NCO was deemed too low (pulse pressure < 15mmHg and/or EtCO_2_ < 14 mmHg [16]).

Echocardiographic monitoring (Affiniti 70 instrument, Philips Healthcare, Suresnes, France) using transthoracic approach was systematically performed in case of VE. Spontaneous echographic contrast (SEC) in cardiac cavities and aortic root was checked in case of low NCO, and categorized according to Fatkin’s definition, then classified as dense if moderate (3+) or severe (4+) [17].

### Study protocol, hemodynamic measurements and FR definition

When inclusion was decided, VA-ECMO tubing was secured by a nurse fully dedicated to the cannulas monitoring, EtCO_2_ was calibrated and echogenicity was confirmed. The ventilator settings, the drugs administration and the pump speed were maintained at constant levels during the protocol period.

The protocol included three sequential steps (PLR-VE test):

1. Baseline: semi recumbent position (45°);
2. PLR test: using an automatic bed elevation technique, the lower limbs were raised to a 30–45° angle while the patient’s trunk was lowered in supine position;
3. VE test: administration of 500 mL of NaCl 0.9% over 15 min after return to baseline position.

Systolic arterial pressure (SAP), mean arterial pressure (MAP), diastolic arterial pressure (DAP), pulse pressure (defined as PP = PAS-PAD), heart rate (HR), central venous pressure, VTI, EtCO_2_ and pump output (PO) were recorded after one minute of stabilization of each step.

Left ventricular outflow tract (LVOT) diameter was measured at the first echocardiography exam. VTI was measured at the level of the LVOT using a 5-chamber view with concomitant electrocardiogram monitoring. Three consecutive measurements (5 in case of atrial fibrillation) were recorded by the same operator without removing the probe from the patient at each step, to calculate a mean VTI value. The intra (5%) and inter (7%)-observer variability of VTI measurements had been tested before the study period (5 VTI measurements had been performed by 3 operators, repeated on 10 different patients).

NCO was calculated as NCO = (LVOT area*VTI*HR)/1000, expressed as L/min. The LVOT area was calculated according to the equation: (π × LVOT diameter ^2^) / 4 in cm^2^. TSO was calculated as TSO = PO + NCO in L/min with PO (L/min) recorded at the time of NCO measurement. Changes (%) of hemodynamic parameters were calculated between baseline and 30 s after PLR initiation (Δx-PLR), and between baseline and the end of VE (Δx-VE).

### Statistical analysis

The study sample size was estimated with the following settings for a single diagnostic test: AUC (for Δ-PLR = 0.75); null hypothesis (AUC = 0.5); power = 0.8; alpha = 0.05, allocation ratio = 0.65. Therefore, 54 measurements had to be recorded. Categorical variables (expressed as absolute values and percentage) were compared using the Chi-squared or the Fisher’s exact test as appropriate. Continuous variables (expressed as medians [25th–75th percentile]) were compared with Student’s t-test or the Mann–Whitney U-test. Linear regression was used to describe the relationship between the VTI variations induced by PLR and VE and the interclass correlation coefficient was calculated. Patients were divided into responders and non-responders according to positive or negative FR which was defined as a VTI increase after VE (ΔVTI-VE) ≥ 15% compared to baseline. The ability of PLR-induced changes in VTI (ΔVTI-PLR) and in EtCO_2_ (ΔEtCO_2_-PLR) to predict FR was evaluated with receiver operating characteristics (ROC) curves and quantified by calculating the area under the curves (AUC) and 95% confidence interval (95% CI). Based on our previous observations showing an increased correlation of EtCO_2_ with lower NCO under VA-ECMO [16], a sub-group analysis was performed according to 3 predefined basal NCO levels (≤ 1, 1–1.5, and >1.5 L/min). Performances of VTI and EtCO_2_ were compared using the ROC AUCs and Delong test. Optimal thresholds were determined by the Youden’s index and other thresholds with better clinical relevance were also studied. Statistical significance was defined as *p* < 0.05. The statistical analyses were performed using R environment (version 4.2.3, R Foundation, Vienna, Austria).

## Results

### Participants, measurements and baseline data

Among 179 patients who received VA-ECMO during the study period, 41 patients were included. Their main characteristics as well as data relative to the ICU outcomes are reported in Table 1.

**Table 1:** Patients characteristics, clinical course and outcomes in intensive care unit

Patients (*n*)	41
*Patients characteristics*	
Male sex, n (%)	33 (80)
Age, years	61 [55–66]
BMI, kg/m²	25.3 [21.4–26.9]
SOFA at ICU admission	11 [9–13]
*Etiology of cardiac failure*	
Acute myocardial infarction, n (%)	25 (61)
Dilated cardiomyopathy, n (%)	8 (20)
Post-cardiotomy, n (%)	4 (10)
Endocarditis, n (%)	3 (7)
Unknown, n (%)	1 (2)
*Context of VA-ECLS implantation*	
VA-ECMO for CS, n (%)	24 (58)
VA-ECMO under CPR, n (%)	17 (42)
*Clinical course in ICU*	
Days under VA-ECMO, days	6 [3–8]
Add of transient left VAD during VA-ECMO, n (%)	5 (12)
Death under VA-ECMO, n (%)	14 (35)
Successful VA-ECMO weaning, n (%)	24 (58)
Heart transplantation or durable left VAD, n (%)	3 (7)
Length of MV, days	7 [4–15]
Renal replacement therapy, n (%)	9 (22)
*Outcomes*	
Length of ICU stay, days	12 [6–22]
ICU-survival, n (%)	23 (56)
6-months survival, n (%)	22 (54)

Values are medians [25th to 75th interquartile ranges] or numbers of patients (n)

BMI: body mass index; SOFA: sequential organ failure assessment score; ICU: intensive care unit; VA-ECMO: veno-arterial extracorporeal membrane oxygenation; CS: cardiogenic shock; CPR: cardiopulmonary resuscitation; MV: mechanical ventilation; VAD: ventricular assist device

**Fig. 1:**
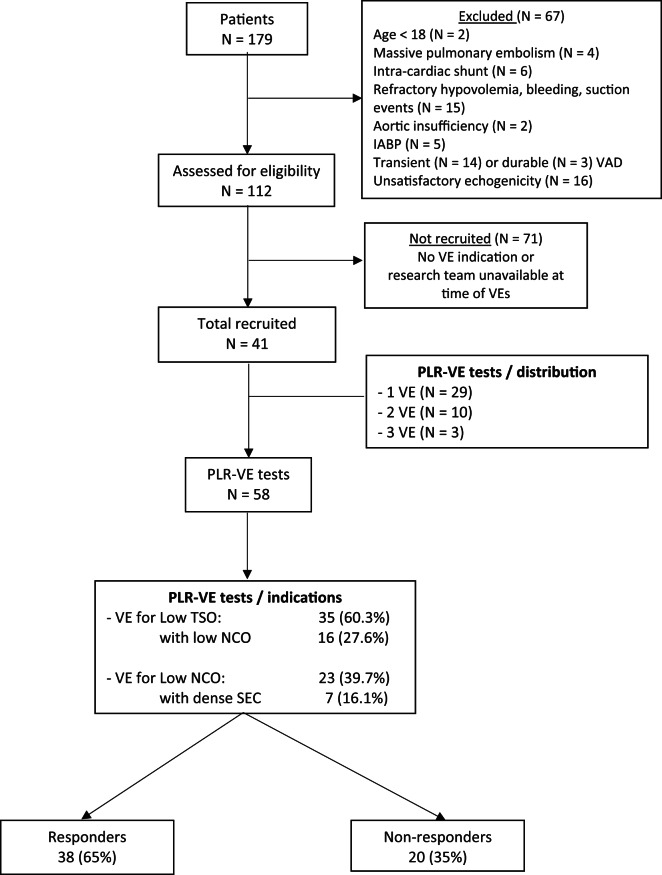
Flow chart. VE: volume expansion; PLR: passive leg raising; TSO: total systemic output; NCO: native cardiac output; VE for Low TSO = VE in order to optimize TSO whatever the NCO; VE for Low NCO = VE in order to optimize NCO while TSO is deemed adapted; IABP: intra-aortic balloon pump support; VAD: ventricular assist device; SEC: spontaneous echographic contrast

Fifty-eight measurements were obtained from these 41 patients (10 patients had 2 PLR-VE tests and 3 patients, 3 tests). VE was realized in 35 (60.3%) cases for Low TSO (of which 16 had associated low NCO) and in 23 (39.7%) cases for Low NCO (of which 7 had dense SEC). No adverse events occurred during the PLR-VE tests; all tests were completed. FR was observed in 38 of the 58 (65%) measurements (Fig. 1).

**Fig. 2:**
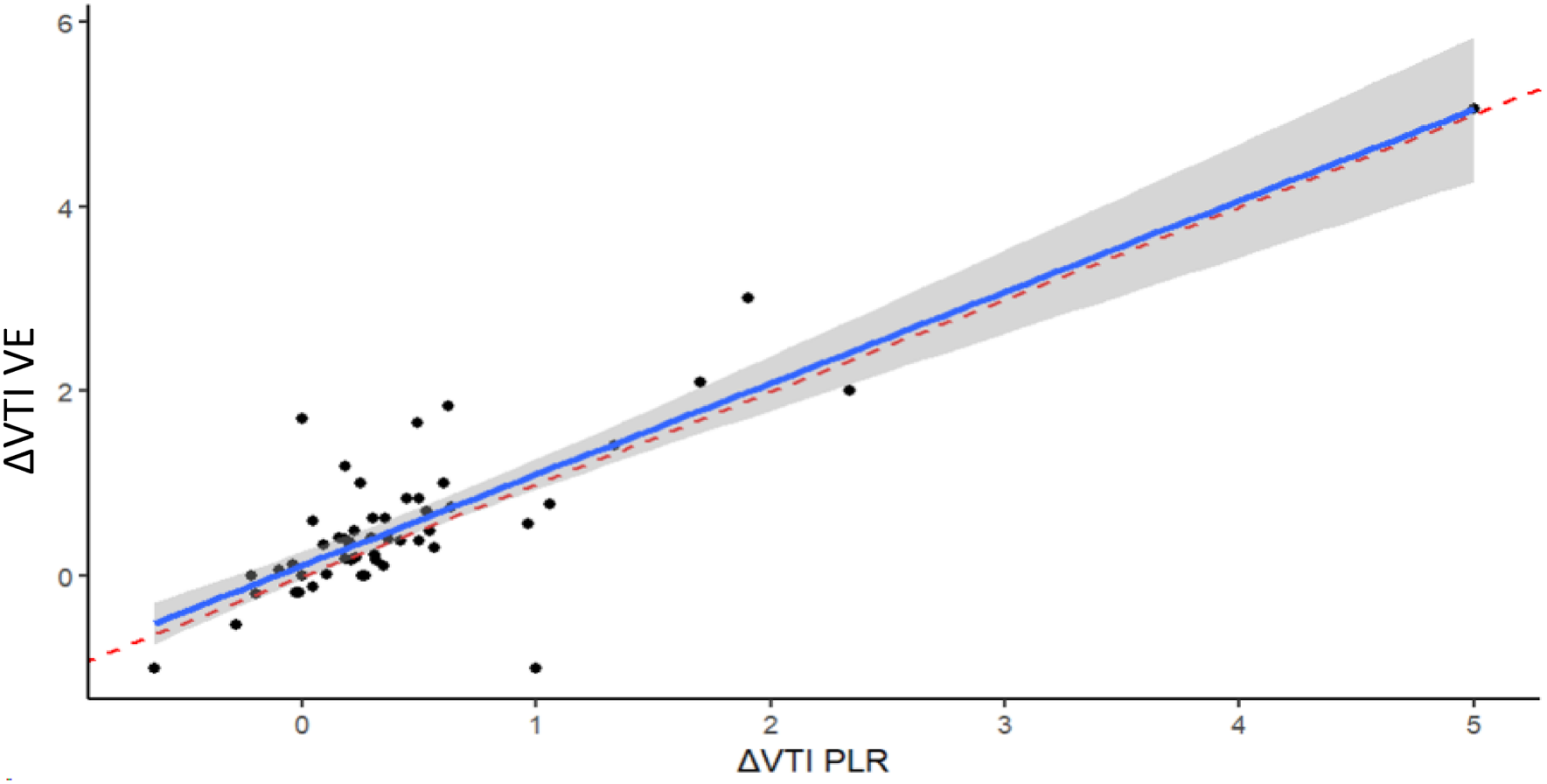
Relationship between ΔVTI induced by passive leg raising (PLR) and ΔVTI induced by volume expansion (VE). Figure consists of linear regression with calculation of intraclass correlation coefficient. The red line represents the bisector. ΔVTI-VE, aortic chamber velocity-time integral variation induced by volume expansion; ΔVTI-PLR, aortic chamber velocity-time integral variation induced by passive leg raising

Table S1 (Additional files) shows that there was no significant statistical difference at baseline between responders and non-responders regarding VE indication, its timing in VA-ECMO course and hemodynamic support. Hemodynamic parameters were not significantly different between responders and non-responders except for heart rate and blood pressure (Table 2). TSO was mainly ensured by PO (median 3.2 L/min, 78% of the TSO). Over all, NCO was low: ≤ 1,5 L/min for 45 (78%) and ≤ 1 L/min for 32 (55%) of cases.

**Table 2:** Hemodynamic parameters at baseline, after PLR and after VE according to fluid responsiveness

Variables	Responders (*n* = 38)	Non-responders (*n* = 20)	*p* value
*HR, beat/min*			
Baseline	104 [86–112]	88 [79–100]	0.04
PLR	104 [83–113]	89 [75–100]	0.06
Volume expansion	100 [83–112]	86 [74–100]	0.04
*SAP, mmHg*			
Baseline	76 [70–88]	84 [79–105]	< 0.01
PLR	93 [80–102]^a^	94 [81–113]	0.50
Volume expansion	95 [87–101]^a^	87 [81–94]	0.06
*DAP, mmHg*			
Baseline	65 [59–70]	75 [69–85]	< 0.01
PLR	71 [65–80]^a^	77 [75–88]	< 0.01
Volume expansion	74 [66–81]^a^	78 [75–82]	0.18
*MAP, mmHg*			
Baseline	68 [63–73]	78 [73–90]	< 0.01
PLR	76 [71–85]^a^	81 [76–93]	0.02
Volume expansion	80 [74–84]^a^	82 [78–85]	0.28
*PP, mmHg*			
Baseline	12 [5–18]	10 [6–28]	0.64
PLR	13 [9–26]	12 [6–26]	0.44
Volume expansion	20 [12–29]^a, b^	8 [5–20]	< 0.01
*EtCO*_2_,* mmHg*			
Baseline	14 [6–19]	11 [6–22]	0.89
PLR	17 [11–23]	12 [5–22]	0.17
Volume expansion	18 [13–24]^a^	11 [5–22]	< 0.01
*CVP, mmHg*			
Baseline	12 [10–15]	12 [10–16]	0.59
PLR	14 [12–17]^a^	14 [12–20]	0.64
Volume expansion	14 [13–19]^a^	13 [11–21]	0.81
*VTI, cm*			
Baseline	2.5 [1.5–4.9]	3 [1.6–4.2]	0.98
PLR	3.8 [2.8–6.6]^a^	2.6 [1.5–4.5]	< 0.01
Volume expansion	4.6 [3.1–8.2]^a^	2.3 [1.4–4.3]	< 0.01
*NCO, L/min*			
Baseline	0.9 [0.5–1.4]	0.9 [0.5–1.3]	0.74
PLR	1.4 [0.8–2.2]^a^	0.8 [0.4–1.5]	0.04
Volume expansion	1.7 [1.0-2.4]^a^	0.7 [0.4–1.4]	< 0.01
*PO, L/min*			
Baseline	3.2 [2.8 3.8]	3.2 [2.6–4.1]	0.68
PLR	3.2 [2.9–3.7]	3.3 [2.6–4.1]	0.66
Volume expansion	3.2 [2.9–3.8]	3.4 [2.7–4.1]	0.45
*TSO, L/min*			
Baseline	4.4 [3.6–5.1]	4.4 [3.7–5.1]	0.80
PLR	4.8 [4.1–5.8]^a^	4.6 [3.8–5.2]	0.16
Volume expansion	5.1 [4.3-6]^a^	4.3 [3.8–5.1]	0.08

Values are medians [25th to 75th interquartile ranges]

HR: heart rate; PLR: passive leg raising; SAP: systolic arterial pressure; DAP: diastolic arterial pressure; MAP: mean arterial pressure; PP: pulse pressure; EtCO_2_: end-tidal carbon dioxide; CVP: central venous pressure; VTI: aortic chamber velocity-time integral; NCO: native cardiac output; PO: pump output; TSO: total systemic output

p value : responders vs. non-responders; ^a^ : *p* < 0.05 vs. baseline; ^b^ : *p* < 0.05 vs. PLR

### Effects of PLR and VE

VTI changes induced by PLR and VE were positively correlated with a high concordance (intraclass correlation coefficient 0.83 [0.73–0.90]) (Fig. 2). In responders, PLR induced significant changes except for heart rate, pulse pressure and pump output whereas VE resulted in significant changes of all parameters except heart rate and pump output (Table 2). Still in responders, PLR-induced changes were significantly lower than those induced by VE for pulse pressure, EtCO_2,_ VTI and NCO (Additional files, Table S2).

### Prediction of fluid responsiveness

**Fig. 3:**
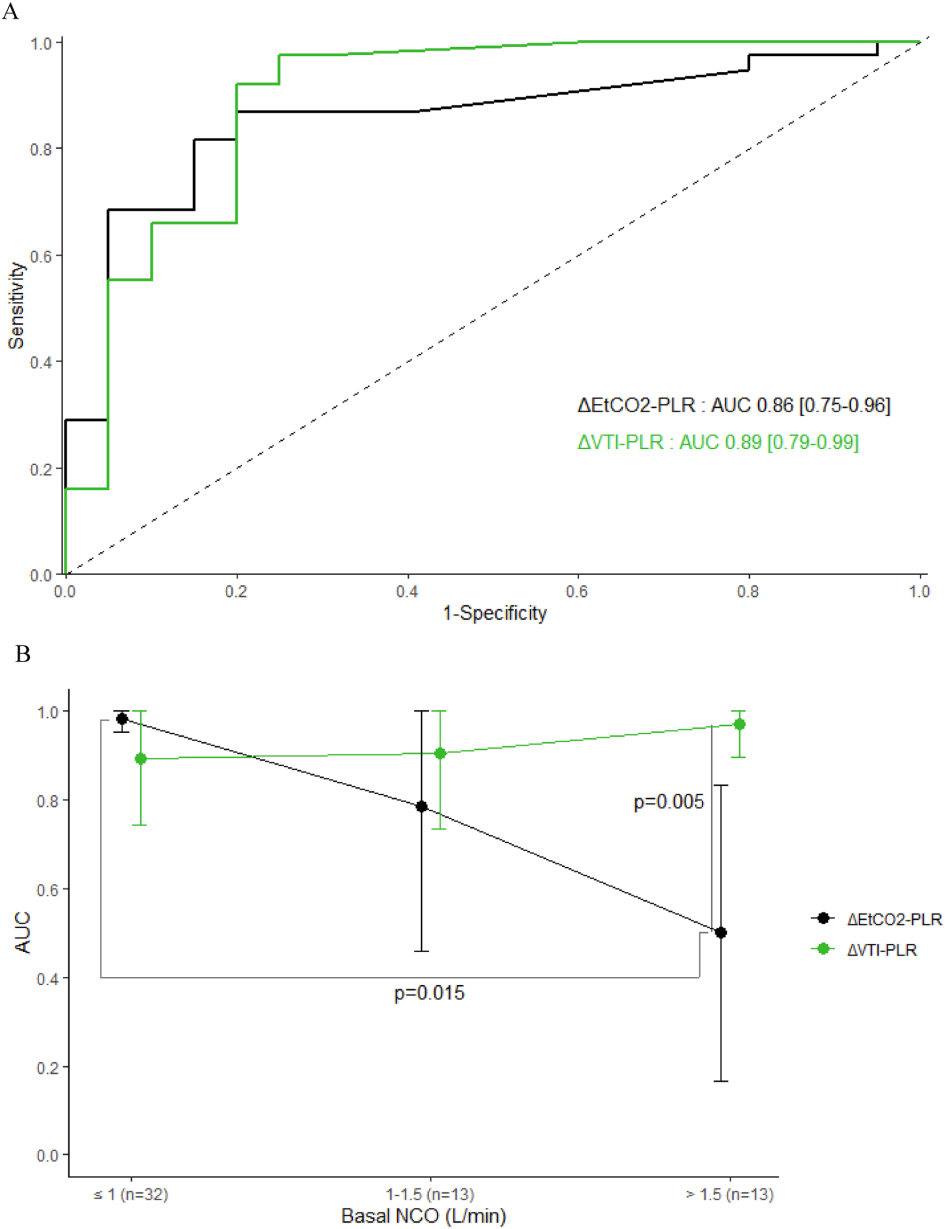
** a** ROC AUCs of ΔVTI and ΔEtCO2 induced by PLR test for predicting fluid responsiveness, in all measurements (n = 58). ROC: receiver operating characteristics curves; AUC: area under the curve; ΔVTI-PLR: aortic velocity-time integral variation induced by passive leg raising test; ΔEtCO2-PLR: end-tidal carbon dioxide variation induced by passive leg raising test.** b** AUCs with their 95% confidence interval of ΔVTI and ΔEtCO_2_ induced by PLR test for predicting fluid responsiveness according to basal NCO. AUC: area under the curve; ΔVTI-PLR: aortic velocity-time integral variation induced by passive leg raising test; ΔEtCO_2_-PLR: end-tidal carbon dioxide variation induced by passive leg raising test. Comparisons of the AUCs between parameters (for each slice of basal NCO) and between slices of NCO (for each parameter) were realized using Delong tests. Only *p* < 0.05 are represented on the figure

ROC AUC for PLR-induced changes in VTI to predict FR was 0.89 [0.79–0.99], with sensitivity, specificity, positive and negative predictive values of 92% [85–100], 80% [69–90], 90% [75–97] and 84% [76–97], respectively, for a threshold of 15% (Fig. 3A). In the sub-group analyses, the ROC AUCs for PLR-induced changes in VTI were similar whether basal NCO was ≤ 1, 1–1.5 or > 1.5 L/min (Fig. 3B).

ROC curve of PLR-induced changes in EtCO_2_ for predicting FR in the all-58 measurements is presented in Fig. 3A. AUC was 0.86 [0.75–0.96] (*p* = 0.592 between ΔEtCO_2_-PLR and ΔVTI-PLR). Figure 3B shows the ROC AUCs of PLR-induced changes in VTI and EtCO_2_ for predicting FR in the subgroup analysis according to basal NCO. The performance of ΔEtCO_2_-PLR to predict FR reached an optimal value when basal NCO was ≤ 1 L/min (ΔEtCO_2_-PLR AUCs when basal NCO ≤ 1 L/min and > 1.5 L/min: 0.98 [0.95-1] vs. 0.50 [0.17–0.83], respectively, *p* = 0.015).

The performances of PLR-induced changes in VTI and EtCO_2_ for predicting FR when basal NCO was ≤ 1 L/min are reported in Table 3.

**Table 3:** Performance of ΔVTI-PLR and ΔEtCO_2_-PLR when native cardiac output ≤ 1 L/min (*n* = 32)

	ΔVTI-PLR	ΔEtCO_2_-PLR
AUC ROC	0.89 [0.74–0.99]	0.98 [0.95–1]
Optimal threshold (%)	18	12
Sensitivity (%)	95 [77–99]	82 [59–94]
Specificity (%)	80 [44–97]	100 [66–100]
PPV (%)	91 [68–99]	100 [78–100]
NPV (%)	89 [56–98]	71 [52–85]

PLR : passive leg raising; ΔVTI-PLR: PLR-induced aortic velocity-time integral variation; ΔEtCO2-PLR: PLR-induced end-tidal carbon dioxide variation; AUROC: area under the receiver operating characteristics curves; PPV: positive predictive value; NPV: negative predictive value

## Discussion

This prospective observational study has evaluated the performance of PLR, and, of EtCO_2_ PLR-induced changes, in predicting FR in VA-ECMO assisted patients. The results show that: 1—NCO changes induced by PLR and by VE were closely related, 2—PLR test is safe under VA-ECMO and can be used to predict FR from VTI changes with a good accuracy whatever basal NCO, 3—In response to PLR, EtCO_2_ changes have a good accuracy to predict FR when basal NCO is ≤ 1 L/min.

Although the presence of a drainage cannula into the inferior vena cava is troublesome, postural manoeuvres that are commonly used to test FR in ICU patients seem promising even in the presence of VA-ECMO [11, 12]. Luo et al. demonstrated that, in VA-ECMO patients with a basal pulse pressure ≥ 15 mmHg, a 10% increasing of VTI during a Trendelenburg manoeuvre was predictive of FR [13]. In our study, we used the PLR test, which has similar effect than the Trendelenburg manoeuvre, but with sustained effects after one minute [11, 18]. PLR test has correctly mimicked VE, with an excellent concordance (Fig. 2) and a discreetly weaker increase in NCO (and related parameters) in responders (Table S2, Additional files) that can be explained by a PLR-induced blood transfer toward to the central compartment inferior to 500 ml [19]. Of note, VE ended after 15 min, before the VE measurements begin, which limited overlapping of the PLR effects on VE [18].

Because thermodilution through a pulmonary artery catheter is not accurate in case of low cardiac output or under VA-ECMO [20], the changes in left ventricle stroke volume were measured using VTI. Changes in the VTI at LVOT during occlusion tests [21], PLR [11, 12] or a mini-fluid challenge [22] for predicting FR has already been studied. Considering the intrinsic and extrinsic variability of echographic measurements, Jozwiak at al. described the conditions for obtaining precise measurements for VTI and identified the smallest change in VTI that can be considered as significant between two examinations [23]. Based on these data, echographic measurements were recorded by the same operator without removing the probe from the patient and 3 ITV measurements (5 in case of atrial fibrillation) were averaged at each step of the PLR-VE test. Interestingly, we observed an intra-observer variability of 5% for 3 VTI measurements performed in sinus rhythm, close from the one found by Jozwiak’s et al. In the same way, we chose a threshold of VTI variation of 15% for studying PLR and VE responsiveness, superior to the least significant change of 11% described by Jozwiak et al. In any cases, although median basal VTI was low (2,4 [1.5–4.9] cm), PLR and VE induced consistent increases in VTI, exceeding 40% (Table S2, Additional files), far superior to the error margin of 11%. Actually, a VE strategy guided by ΔVTI-PLR >15% would have avoided 16 out of 20 inadequate VE at the cost of missing 3 adequate VE out of 38 in this study.

In clinical practice, VTI catching may be challenging and time consuming in VA-ECMO patients with low pulsatility, when electrocardiogram synchronization, transoesophageal echography or repeated measures are needed to improve the measurement quality. Consequently, surrogates for ΔVTI to assess FR would be appreciated, provided that they are effective, reliable, and easier to use. EtCO_2_ has already been described as a reliable estimate of pulmonary blood flow. Experimental [24] and clinical [25] data in non-ECMO conditions described the respective role of the variations in PaCO_2_ (CO_2_ delivery to the lung) and alveolar dead space (effective alveolar ventilation) in ΔEtCO_2_ during non-steady state situations. In the context of VA-ECMO, gas exchange is partitioned between the lungs and the extracorporeal membrane and their respective ventilation-to-perfusion ratios (V/Q). In our study, VA-ECMO V/Q was maintained constant (constant pump output and sweep gas flow) as well as the ventilation settings during the PLR-VE tests. Therefore, the EtCO_2_ increase in responders might have resulted from reductions of the alveolar dead space (through pulmonary flow increases); that were greater as the basal NCO was low. These results are in agreement with a previous publication in which we observed high correlation of EtCO_2_ with NCO < 2 L/min [16] and with an experimental model of VA-ECMO, where Bachmann and co-workers showed that elimination of CO_2_ from the lungs followed the increase of pulmonary blood flow (between 0 and 2 L/min) during VA-ECMO flow reduction [26]. The limitation of EtCO_2_ monitoring is related to the access to exhaled CO_2_ that, in order to be trustable, requires that the patient be intubated.

Finally, whatever its indication, VE resulted in increasing TSO only through an increased NCO. Indeed, after having excluded situations of obvious hypovolemia, we observed that, at constant ECMO setting, PO remained stable after VE, whatever FR. VE was likely to increase venous return to the right heart, and to improve transpulmonary flow provided there was a preload dependence. Interestingly, we observed that NCO increase was sufficient to lead to a significant increase in TSO (ΔTSO >15%) in 19 out of 38 (50%) cases without difference according to VE indication (*p* = 0.1) or the presence or not of basal NCO ≤ 1 L/min (*p* = 0.65). VE has also been realized to optimize NCO, independently from a TSO deemed adapted. The decision of VE in this situation (39.7% in this study) was motivated by preventing the harmful consequences of blood stasis in pulmonary circulation, cardiac cavities and aortic root [4]. Even though the potential benefits of maintaining a minimal NCO [27] could be thwarted by the risk of an increased fluid imbalance [9]– [10], VE was decided mostly in the absence of dense SEC (*n* = 16, 70%) [5].

### Study limitations

Our study has several limitations. First, it is a single-centre study, which may limit generalizability across different clinical settings. Second, we used the same baseline measurement for both PLR and VE. This introduces a risk of regression to the mean phenomenon and also of overlapping effects of the PLR on VE, which could have artificially inflated the apparent correlation between these interventions and overestimate diagnostic accuracy. Third, although the variations of EtCO2 are mainly based on variations of alveolar dead space at low NCO level, a PaCO2 maintained in normal range with a ventilation to perfusion ratio at ECMO around 1 might be respected for the reproducibility of our results. Fourth, since FR was only tested at the end of VE, this study does not provide information on transient and persistent effects in the responders patients [28]. Fifth, EtCO_2_ changes cannot be used in nonventilated patients. Finally, in our study, VE for “Low TSO” without response on NCO were considered inadequate, what could be debatable in clinical practice if it allows to increase the pump speed and PO to reach an adapted TSO.

## Conclusion

In conclusion, PLR can predict FR under VA-ECMO. EtCO_2_ changes induced by PLR can be used to detect FR in ventilated patients with NCO ≤ 1 L/min under VA-ECMO. This test is easy to perform, reliable and may help to avoid unnecessary and potentially harmful fluid loading.

## Supplementary Information

Below is the link to the electronic supplementary material.

Supplementary Material 1

## Data Availability

The datasets generated and/or analysed during the current study are not publicly available due to non-anonymized datas but are available from the corresponding author on reasonable request.

## Abbreviations

VA-ECMO: Veno-arterial extracorporeal membrane oxygenation
EtCO_2_: End-tidal carbon dioxide
AUC: Area under the curve
PO: Pump output
NCO: Native cardiac output
TSO: Total systemic output
VE: Volume expansion
PLR: Passive leg raising
FR: Fluid responsiveness
VTI: Aortic chamber velocity-time integral
ICU: Intensive care unit
PP: Pulse pressure
SEC: Spontaneous echographic contrast
SAP: Systolic arterial pressure
MAP: Mean arterial pressure
DAP: Diastolic arterial pressure
HR: Heart rate
LVOT: Left ventricular outflow tract
ICC: Intraclass correlation coefficient
ROC: Receiver operating characteristics
