# Supplementary material for: End-tidal carbon dioxide changes induced by passive leg raising can predict fluid responsiveness in patients on veno-arterial extracorporeal membrane oxygenation: a prospective, interventional study
Source: Ann Intensive Care. 2025 Nov 20;15:185. doi: 10.1186/s13613-025-01604-2 (PMC12630523; doi:10.1186/s13613-025-01604-2)
Supplement: Supplementary file 1 — Supplementary Material 1 [file 13613_2025_1604_MOESM1_ESM.docx]

**Additional files**

Figure S1

Low TSO

Low NCO

(🗹 TSO)

Increase PO

(↑PS)

Decrease PO

(↓PS)

Low TSO

🗹 TSO

Low NCO

(🗹 TSO)

Low NCO

(🗹 TSO)

🗹 NCO

No further action

**Volume Expansion**

🗹 TSO

🗹 NCO

Low NCO

Low TSO

Increase PO PO

LV venting

🗹 TSO

🗹 NCO

No further action

🗹 NCO

(🗹 TSO)

Inotrope

**Title :** Local protocol for managing Low TSO and Low NCO during VA-ECMO.

**Legend**

TSO was deemed too low in case of hypotension (defined as MAP < 65mmHg) and/or signs of hypoperfusion (skin mottling, oligoanuria defined as diuresis < 0.5 mL/kg/h, lactate > 2.2mmol/L). NCO was deemed too low in case of pulse pressure < 15mmHg and/or end-tidal carbon dioxide < 14 mmHg [16].

The optimization of TSO and NCO was based on a three-steps process: 1/ VA-ECMO’s pump output optimization through pump speed adaptation. 2/ VE test to increase NCO (for “Low TSO” or “Low NCO”) or even to allow an additional pump speed increase (for Low TSO only), 3/ the modification of the amine therapy regimen (for Low TSO or Low NCO) or the use of left ventricular venting (for Low NCO only).

TSO, total systemic output; NCO, native cardiac output; 🗹, TSO or NCO deemed adapted; PO, pump output; PS, pump speed; LV: left ventricular; VA-ECMO, veno-arterial extracorporeal membrane oxygenation.

Table S1

| Table S1: Indication and timing of volume expansion, respiratory and hemodynamic support according to fluid responsiveness. | | | |
| --- | --- | --- | --- |
| Measures (n) | **Responders (n = 38)** | **Non-responders (n = 20)** | **p** |
| **Indication and timing of VE** |  |  |  |
| VE for Low TSO/VE for Low NCO | 22/16 | 13/7 | 0.59 |
| Delay from VA-ECMO implantation to VE, days | 1.5 [1-2] | 1 [1-2] | 0.92 |
| **Low NCO before VE, n (%)** | 26 (68) | 13 (65) | 0.79 |
| **Laboratory variables** |  |  |  |
| Lactate, mmol/L | 3.7 [1.8-6.4] | 2.8 [1.7-5.9] | 0.49 |
| ScvO_2_, % | 76 [64-82] | 79 [74-84] | 0.06 |
| PaCO_2_, mmHg | 36 [30-38] | 35 [33-40] | 0.57 |
| **Ventilatory settings** |  |  |  |
| Tidal volume, mL | 400 [340-425] | 400 [355-450] | 0.37 |
| Respiratory rate, breaths/min | 12 [12-14] | 14 [12-15] | 0.06 |
| Ventilator minute volume (V lung), L/min | 4.8 [4-6.3] | 5.9 [4.3-6.5] | 0.25 |
| PEEP, mmHg | 8 [7-12] | 9 [8-10.5] | 1 |
| **Hemodynamic support** |  |  |  |
| VA-ECMO settings |  |  |  |
| Pump rotation speed, round/min | 2491 [2305-2786] | 2582 [2392-2945] | 0.48 |
| VA-ECMO flow (Q ECLS), L/min | 3.2 [2.8-3.8] | 3.2 [2.7-4.1] | 0.68 |
| VA-ECMO sweep gas flow (V ECLS), L/min | 3.7 [2.5-4.6] | 4 [3-5.6] | 0.2 |
| V/Q ECMO | 1.2 [0.9-1.6] | 1.2 [0.9-1.7] | 0.36 |
| V lung/V ECMO | 1.5 [1-2] | 1.3 [0.9-2] | 0.64 |
| Vasopressors and inotropes |  |  |  |
| Inhaled nitric oxide, n (%) | 2 (5) | 2 (10) | 0.6 |
| Norepinephrine, n (%) | 36 (95) | 17 (85) | 0.32 |
| Dose, µg/kg/min | 0.48 [0.20-0.79] | 0.79 [0.46-0.86] | 0.14 |
| Dobutamine, n (%) | 21 (55) | 9 (45) | 0.45 |
| Dose, µg/kg/min | 4.99 [2.81-6.77] | 7.73 [3.28-9.96] | 0.22 |
| **Values are medians [25^th^ to 75^th^ interquartile ranges] or numbers of patients (n).**  **Low total systemic output was defined as mean arterial pressure < 65mmHg and/or signs of hypoperfusion (skin mottling, diuresis < 0.5 mL/kg/h, lactate > 2.2 mmol/L), low native cardiac output as pulse pressure < 15 mmHg and/or EtCO_2_ < 14 mmHg**  **VA-ECMO: veno-arterial extracorporeal membrane oxygenation; VE: volume expansion; TSO: total systemic output; NCO: native cardiac output; Low TSO: mean arterial pressure < 65mmHg and/or signs of hypoperfusion (skin mottling, diuresis < 0.5 mL/kg/h, lactate > 2.2 mmol/L); Low NCO: PP < 15 mmHg and/or EtCO_2_ < 14 mmHg; ScvO_2_: central venous oxygene saturation; PEEP: positive end expiratory pressure; V/Q: ventilation-to-perfusion ratio** | | | |

Table S2

| Table S2: Hemodynamic variations after passive leg raising and after volume expansion compare to baseline in responders (n=38). | | | | |
| --- | --- | --- | --- | --- |
|  | PLR vs baseline (%) | VE vs baseline (%) | VE vs PLR (%) | VE vs PLR (p) |
| ΔHR, bpm | 0 [-4/0] | -3 [-7/0] | -1 [-4/+1] | 0.07 |
| ΔCVP, mmHg | +18 [+9/+25] | +27 [+8/+40] | +9 [-7/+17] | 0.17 |
| ΔSAP, mmHg | +15 [+4/+26] | +20 [+9/+34] | +4 [-4/+16] | 0.1 |
| ΔMAP, mmHg | +13 [+5/+21] | +15 [+4/+26] | +1 [-6/+10] | 0.51 |
| ΔDAP, mmHg | +9 [+5/+19] | +11 [+2/+22] | -3 [-6/+8] | 0.9 |
| ΔPP, mmHg | +35 [+10/+95] | +67 [+37/+190] | +30 [0/+61] | < 0.01 |
| ΔEtCO_2,_ mmHg | +24 [+8/+59] | +36 [+11/+80] | +4 [-5/+29] | < 0.01 |
| ΔVTI, cm | +42 [+20/+61] | +57 [+38/+100] | +11 [-4/+24] | < 0.01 |
| ΔNCO, L/min | +38 [+18/+61] | +49 [+31/95] | +14 [-5/+25] | < 0.05 |
| ΔPO, L/min | 0 [-2/+2] | 0 [-2/+3] | 0 [-1/+2] | 0.27 |
| ΔTSO, L/min | +9 [+5/+16] | +15 [+9/+20] | +3 [-1/+12] | < 0.01 |
| **Values are medians [25^th^ to 75^th^ interquartile ranges]**  **PLR: passive leg raising; VE: volume expansion; HR: heart rate; CVP: central venous pressure; SAP: systolic arterial pressure; DAP: diastolic arterial pressure; MAP: mean arterial pressure; PP: pulse pressure; EtCO2: end-tidal carbon dioxide; VTI: aortic chamber velocity-time integral; NCO: native cardiac output; PO: pump output; TSO: total systemic output.** | | | | |
